# The effects of sleep disturbances on patients receiving methadone treatment for opioid use disorder: cortical thickness evidence

**DOI:** 10.3389/fpsyt.2026.1688680

**Published:** 2026-03-25

**Authors:** Feng Cui, Yan Chang, Xin-Wei Ma, Tang-Fen Wang, Xin Zhang, Tao Hu, Qing-Qian Guo, Xiao-Yu Feng, Xu Guo, Tian-Xiao Shen, Xiao-Dong Yang, Jian Zheng, Ke-Jia Hu, Jian-Bing Zhu

**Affiliations:** 1 School of Biomedical Engineering (Suzhou), Division of Life Science and Medicine, University of Science and Technology of China, Hefei, Anhui, China; 2 Suzhou Institute of Biomedical Engineering and Technology, Chinese Academy of Sciences, Suzhou, Jiangsu, China; 3 Ji Hua Laboratory, Foshan, Guangdong, China; 4 Suzhou Hospital, Affiliated Hospital of Medical School, Nanjing University, Suzhou, China; 5 Department of Neurosurgery, Center for Functional Neurosurgery, Ruijin Hospital, Shanghai Jiao Tong University School of Medicine, Shanghai, China; 6 Department of Neurosurgery, The First Affiliated Hospital of Wannan Medical College (Yijishan Hospital of Wannan Medical College), Wuhu, Anhui, China

**Keywords:** cortical thickness, sleep disturbances, methadone maintenance treatment, surface-based morphometry, opioid use disorder

## Abstract

**Background:**

Sleep disturbances are prevalent among patients with methadone maintenance treatment (MMT) for opioid use disorder (OUD), yet the neurobiological implications remain poorly understood. So we investigated cortical thickness between the MMT and the MMT with sleep disturbances (MMTS) patients to determine the interplay between MMT, sleep disturbances, and cortical remodeling.

**Methods:**

This study was approved by the ethics committee of the Affiliated Suzhou Hospital, Medical School of Nanjing University (IRB2024070). We recruited 30 MMT and 33 MMTS participants in this study. All subjects underwent a high-resolution MRI for calculating the cortical thickness by the surface-based morphometry (SBM). Meanwhile, sleep quality of all subjects were recorded using the Pittsburgh Sleep Quality Index (PSQI) and Insomnia Severity Index (ISI), and evaluated the relationship between the cortical alternations and sleep disturbances.

**Results:**

There were significant increases of cortical thickness in MMTS patients within the left entorhinal cortex, right pericalcarine cortex, and left frontalpole cortex compared to the MMT patients after adjusting for covariates (all *p* < 0.05). The increase thickness of the left frontalpole cortex was positively correlated with the PSQI (*p* = 0.038, r=0.27) and its sub-items (Sleep Quality, *p* = 0.04, r=0.27; Daytime Dysfunction, *p* = 0.037, r=0.28). While the increase thickness of the left entorhinal cortex (Sleep Quality, *p* = 0.017, r=0.31; Sleep Duration, *p* = 0.041, r=0.27) and right pericalcarine coetex(Sleep Latency, *p* = 0.036, r=0.27) showed associations with specific sleep items. No correlations emerged with the ISI.

**Conclusions:**

These findings identify increased cortical thickness in the left entorhinal, right pericalcarine, and left frontalpole cortices in MMTS patients, potentially driven by the receptor effects of methadone, neuroinflammation, or synaptic plasticity linked to sleep disturbances. The study underscored sleep disturbances as a critical modifier of neuroanatomical alternations in MMT, advocating for sleep-targeted interventions to mitigate relapse risk and optimize MMT strategies. Longitudinal studies integrating objective sleep measures were warranted to validate these biomarkers for clinical prognostication.

## 1 Introduction

Opioid addiction remains a significant global public health challenge. In 2022, around 60 million individuals worldwide used opioids, accounting for 1.2% of the global population, and at least 7 million of them engaged in the law for drug-related offenses according to the 2024 world drug report from the United Nations Office on Drugs and Crime (UNODC). Although policy and pharmacological interventions, such as naloxone, buprenorphine, and methadone maintenance treatment (MMT), have reduced the rate of opioid abuse in certain regions, the long-term management of opioid dependence remains difficult (1). Moreover, MMT patients may discontinue treatment due to potential side effects or inadequate efficacy, which could become an important predictor of their prognosis (2).

Methadone, the most commonly used medication for opioid addiction treatment in China, is a long-acting μ-opioid receptor agonist (3). It suppresses withdrawal symptoms and cravings by mimicking opioid effects, serving as a core strategy to curb illegal drug consumption (3). However, due to the unique pharmacokinetic properties of methadone, such as a long half-life and individual metabolic differences, it may cause certain adverse effects, especially sleep disturbances, after central nervous system (CNS) action (4). A small-scale report indicate that over 78% of MMT patients complain of poor sleep quality, manifesting as difficulty falling asleep, fragmented sleep, and daytime functional impairment (5, 6). A polysomnography(PSG) study further confirm that MMT patients with sleep disturbances (MMTS) exhibit reduced slow-wave and prolonged Rapid Eye Movement (REM) latency in their sleep (7). Evidence suggests that sleep disturbances may not only be regarded as a secondary symptom of the MMT effects, but also an independent risk factor in driving substance-use relapse (5). Therefore, sleep disturbances should not be ignored in MMT patients.

Neuroimaging studies have gradually revealed that sleep disturbances are closely related to structural brain changes (8). For example, research demonstrates the thinning of the prefrontal cortex (9, 10), anterior cingulate cortex (11) as well as hippocampus atrophy in chronic insomnia patients (12, 13). Interestingly, some regions are not only implicated in sleep regulation but also constitute core nodes of the addiction circuitry (14). However, previous studies of MMT patients have ignored the sleep quality as a major covariate and primarily focused on addiction itself. Therefore, this methodological limitation may lead to a potential bias, making it difficult to accurately distinguish the independent or interactive effects of “addiction-treatment-sleep”. Whether sleep - related cortical remodeling exhibits specific patterns in MMT patients and whether it can serve as a biomarker to predict treatment adherence or relapse risk remains unsupported by evidence.

Our study may represent the initial exploration of differences in cortical thickness between the MMTS and MMT patients. We hypothesize that sleep disturbance could accelerate cortical remodeling in specific brain regions of MMT patients, effectively distinguishing between the two MMT subgroups and providing quantitative indicators of sleep-related brain impairment. Therefore, the current research aims to both elucidate the contribution of sleep disturbances in the pathology of opioid dependence during MMT and provide imaging evidence for early identification of the high-risk MMTS patients and development of sleep-targeted intervention strategies. Ultimately, it optimizes treatment efficacy and improves quality of life of MMTS patients.

## 2 Materials and methods

### 2.1 Participants

This prospective study was conducted in accordance with the Helsinki Declaration and approved by the ethics committee of the Affiliated Suzhou Hospital, Medical School of Nanjing University (IRB2024070), Clinical trial number: not applicable. A total of 30 MMT patients and 33 MMTS patients were recruited by the Affiliated Suzhou Hospital, Medical School of Nanjing University in outpatient clinics and local communities between July 2023 and December 2024. According to the Diagnostic and Statistical Manual of Mental Disorders-V for Substance Use Disorder (SUD), the MMT was diagnosed by experienced addiction specialists and ensured that each participant was receiving or had received standard MMT treatment. The exclusion criteria were as follow: (a) More than one abnormalities in blood and urine indicators in physical examination. (b) Mental disorders such as depression, epilepsy, Alzheimer’s disease, Parkinson’s disease, schizophrenia or severe cognitive. (c) Previous CNS impairments, such as cerebral infarction, cerebral hemorrhage, brain tumor and brain trauma. (d) Contraindications for magnetic resonance imaging (MRI), such as the presence of pacemakers, non - removable dental appliances, or a history of claustrophobia. The sample size of this study was calculated on the basis of a “*Post hoc*” power analysis with G Power software [effect size f(U) = 0.4; a err prob = 0.05; power (1-b err prob) = 0.88]. It revealed that a sample size of at least 30 patients would have a 88% power to detect such a difference as statistically significant at a level (a) of 0.05 in this study. All the participants were right-handed (determined by self-report) to minimize potential confounding effects of brain lateralization, and had signed the informed consent.

### 2.2 Sleep disturbances assessments

All participants completed two sleep quality tests, including the Pittsburgh Sleep Quality Index questionnaire (PSQI) and Insomnia Severity Index (ISI). The PSQI was employed to evaluate the sleep quality of each participant over the past month, covering 7 aspects or sub items such as sleep duration, disturbances and daytime dysfunction. The ISI was utilized to assess the severity of insomnia symptoms, including difficulties in falling asleep and staying asleep. Generally, a PSQI score greater than 4 and an ISI score greater than 6 are commonly recognized as indicative of sleep disturbances. To ensure consistency and reliability, all tests were administered to all participants by the same experienced professional.

### 2.3 Data acquisition

All participants underwent a MRI scanning by a 3T system (GE Discovery 750w 3.0T), which was fitted with a 32 channel head-neck coil. They were instructed to stay calm, keep their eyes shut, and avoid any movement throughout the data acquisition. Axial T1/T2-weighed images, T2 fluid-attenuated inversion recovery (T2-FLAIR) images and 3D Time-Of-Flight Magnetic Resonance Angiography(3D TOF-MRA) images were obtained to exclude those participants with significant structural brain abnormalities. Main data were collected with a high - resolution 3D Fast Spoiled Gradient - Recalled Echo sequence (FSPGR) sequence, the protocol was follow: repetition time (TR)/echo time (TE) of 8.7/3.2ms, a slice thickness of 1.0 mm, a field of view (FOV) measuring 256 mm × 256 mm, a matrix size of 256 × 256, a flip angle of 12°, 160 slices with no gap, and a scan duration of 4 minutes and 23 seconds. The sections were parallelized to the line connecting the anterior and posterior commissures.

### 2.4 Surface-based morphometry and Region of Interest analysis

The SBM analysis was conducted with SPM12 (https://www.fil.ion.ucl.ac.uk/spm/) and CAT12 (https://neuro-jena.github.io/cat/) software. The performance of CAT12 in structural MRI processing was comparable to Freesurfer, especially in terms of accuracy and reliability in cortical segmentation and morphological measurement, with high consistency (15). Firstly, the FSPGR images of each participant were pre-processed, including bias correction (16), tissue segmentation, and normalization (17) to the standard template space using the DARTEL method (18). After th tissue segmentation progress, a necessary quality-check was critical to ensure the accuracy of the segmentation results. Secondly, the cortical surfaces were extracted and inflated to facilitate the comparison of cortical thickness across subjects. Then, the normalized cortical thickness was obtained by estimating the distance between the white matter and gray matter boundaries, and the cortical thickness within all ROIs was extracted from a total of 68 bilateral cortical structures defined by the Desikan-Killiany atlas (19). This study adopted an exploratory approach covering all 68 cortical regions defined by the atlas. Finally, statistical analysis was performed to identify the regions with significant differences in cortical thickness between groups. The total intracranial volume (TIV) was also calculated and used as a covariate to correct for the effects of head size differences among subjects.

### 2.5 Statistical analysis

In this study, all demographic and cortical thickness data were processed by the SPSS software version 26.0 (SPSS, Chicago, IL, USA). For the demographic data, an unpaired two - sample t - test was conducted to analyze continuous variables. A chi - square test was applied to categorical variables, while a nonparametric Mann–Whitney U test was used for variables that did not follow a normal distribution. All statistical tests were two - sided, and a ***p* <** 0.05 was considered statistically significant. Continuous variables were presented as mean ± standard deviation (SD), and other variables were expressed as medians ± interquartile ranges (IQR). In order to determine the cortical thickness difference between MMT and MMTS patients, an analysis of covariance (ANCOVA) was performed. The normalized cortical thickness of each cortical region was analyzed, with age, gender, educational duration, and the TIV being regarded as covariates, a ***p* <** 0.05 was regarded as statistically significant as well. Moreover, a partial correlation analysis was conducted to investigate the correlations between the cortical thickness differences and the sleep characteristics, with the above 4 factors considered as covariates.

## 3 Results

### 3.1 Demographic characteristics

The demographic characteristics of all subjects were shown in **Table 1**. There was no significant difference in age, gender, education duration, handedness, smoking, alcoholism and medication history between the MMT and MMTS patients (***p***>0.05). Besides, participants of the MMT demonstrated PSQI scores below 6, with an average of 3 and their ISI scores were all under 7, averaging 10. In contrast, other participants of the MMTS exhibited PSQI scores above 6, with an average of 15 and their ISI scores were all above 7, averaging 15. Therefore, there is a significant statistical difference in PSQI (including 7 sub items) and ISI scores between the MMT and the MMT patients (***p* <** 0.001).

**Table 1:** Demographic and clinical characteristics of the MMT and MMTS patients.

Characteristics	MMT (n=30)	MMTS (n=33)	t/χ²	P
Age (years)	45.3 ± 8.2	47.1 ± 7.9	1.06	0.38
Gender (Male/Female)	22/8	24/9	1.28	0.26
Education (years)	10.5 ± 2.8	9.8 ± 3.1	-0.98	0.42
TIV (mm^3^)	1434. 7 ± 104. 5	1470.4 ± 130.4	-1.20	0.24
Handedness (Right/Left)	30/0	33/0	–	–
Smoking (Yes/No)	18/12	20/13	<0.01	0.96
Alcoholism (Yes/No)	10/20	12/21	0.08	0.78
PSQI	3.0 ± 1.2	15.0 ± 3.5	-9.96	<0.001*
ISI	5.0 ± 1.8	15.0 ± 4.2	-16.15	<0.001*
Medication history(Yes/No)	5/25	9/24	2.09	0.35

All data presented as mean ± SD or counts. MMT, Methadone Maintenance Treatment; MMTS, MMT with sleep disturbances; TIV, total intracranial volume; PSQI, Pittsburgh Sleep Quality Index; ISI, Insomnia Severity Index. The Medication history was only the statistical situation of the past two years. Bold p indicated statistical significance.The * symbol indicates statistical significance at p < 0.05.

### 3.2 Cortical thickness difference in ROIs

**Figure 1:**
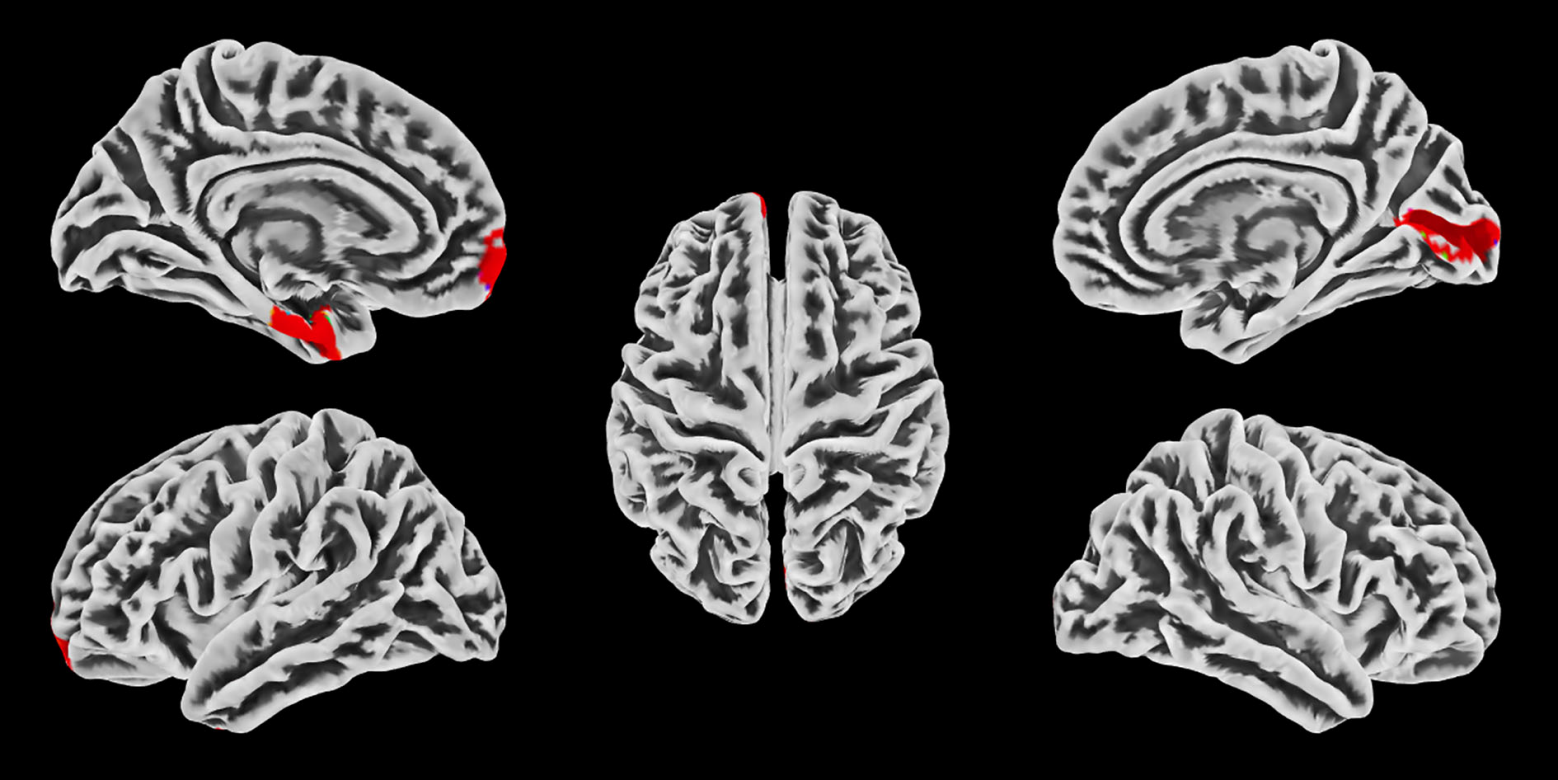
SBM and ROI analysis between the MMT and MMTS patients shows significant increase of cortical thickness in the left entorhinal cortex, right pericalcarine cortex and the left_frontalpole cortex of the MMTS patients (red regions, p<0.05). Cortical images are displayed in five views (Left to Right and top to bottom: left lateral view, right lateral view, top view, left medial view and right medial view).

**Figure 2:**
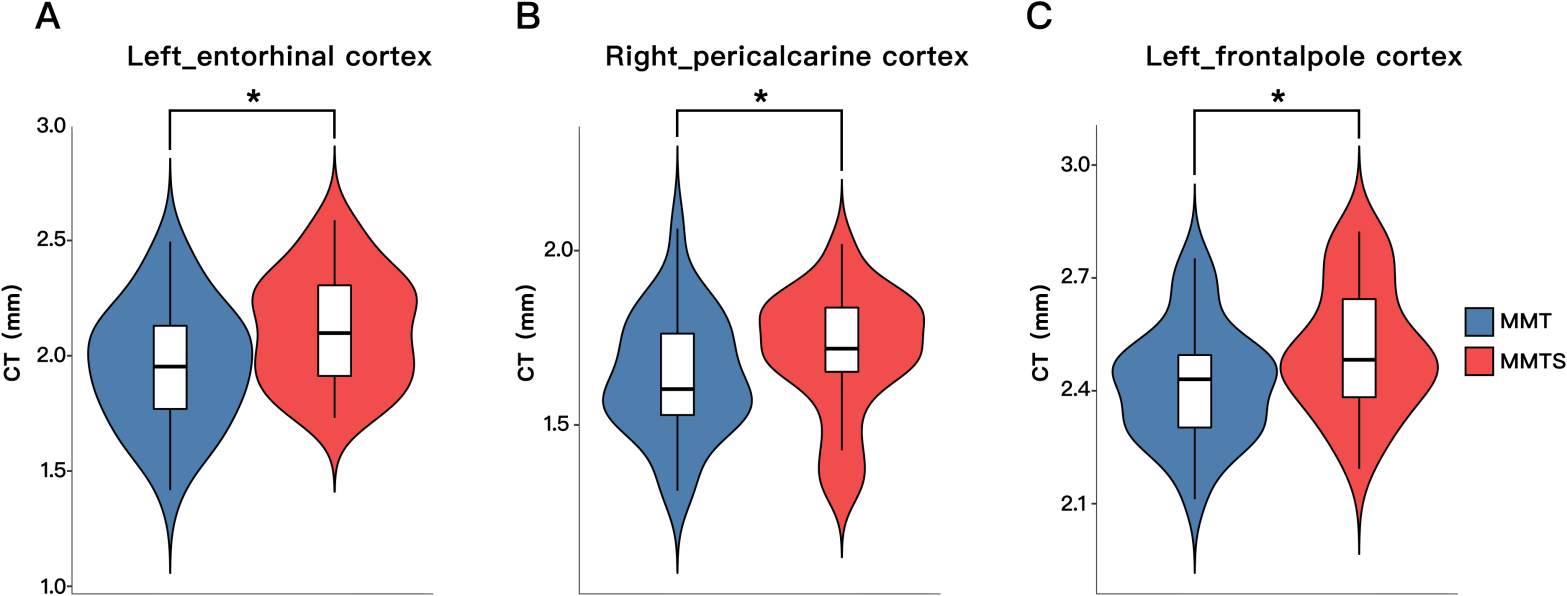
Cortical thickness differences between the MMT and MMTS patients. These results were adjusted for age, gender, education duration, and total intracranial volume. MMT, Methadone Maintenance Treatment; MMTS, MMT with sleep disturbances. **(A)** Left entorhinal cortex. **(B)** Right pericalcarine cortex. **(C)** Left frontalpole cortex. * indicated statistical significance.

Statistical results for normalized thickness of the significant cortical thickness difference between the MMT and MMTS patients had been showed in **Figures 1**, **2**. An ANCOVA analysis revealed that, even after adjusting for 4 covariates, compare with the MMT patients, participants of the MMTS had significantly increased cortical thickness in three regions (left entorhinal cortex, 1.96 ± 0.26mm vs 2.12 ± 0.24mm, F = 7.09, ***p*** = 0.01; right pericalcarine cortex, 1.64 ± 0.17mm vs 1.71 ± 0.18mm, F = 4.14 ***p*** = 0.047;and left_frontalpole cortex, 2.44 ± 0.21mm vs 2.51 ± 0.17mm, F = 4.10, ***p*** = 0.047).

### 3.3 Correlations between the significantly increased cortical thickness and sleep characteristics

**Figure 3:**
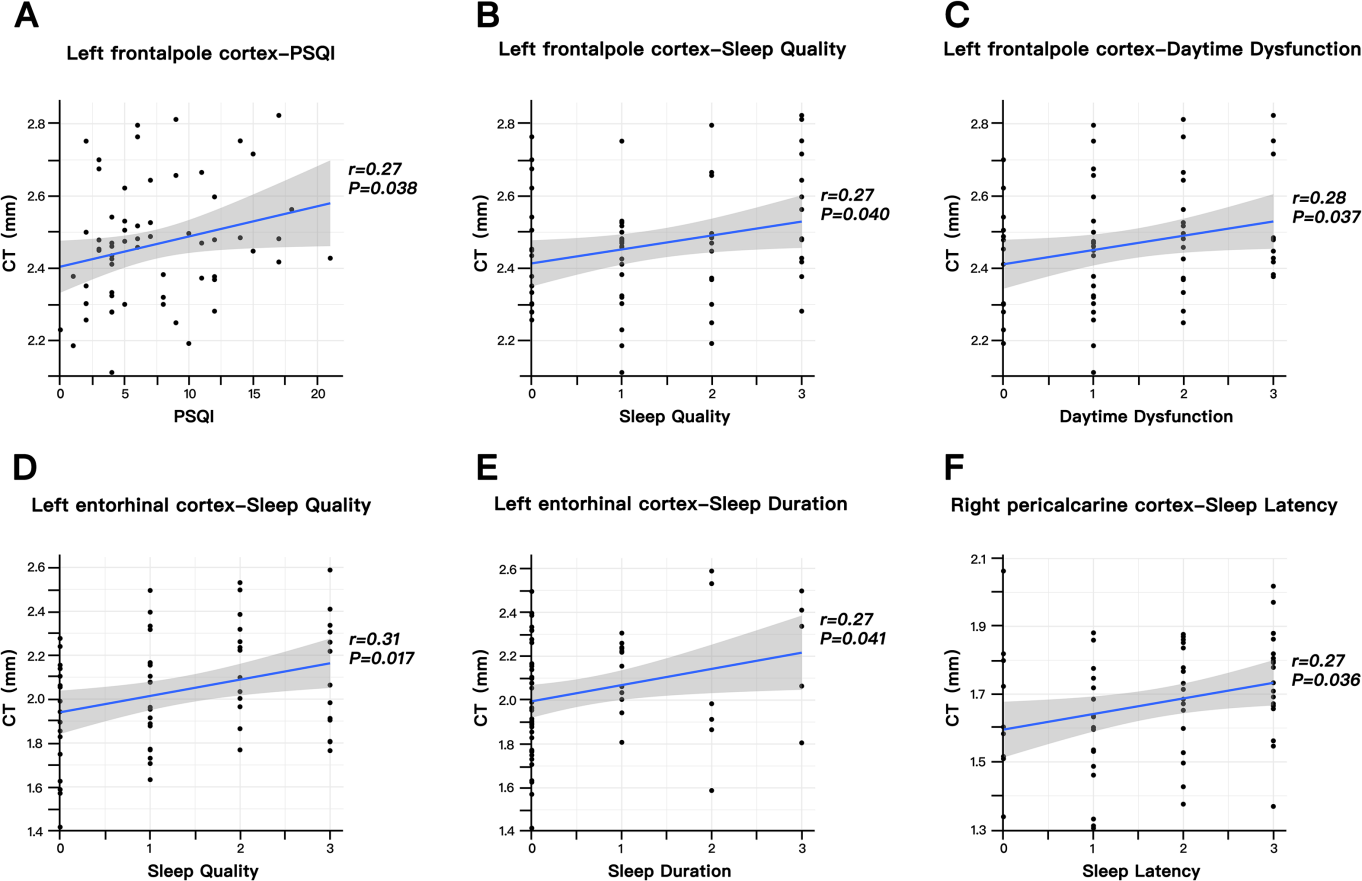
Correlations between significant increase of cortical thickness and sleep characteristics in the MMTS patients. Partial correlation adjusted for age, gender, education duration, and total intracranial volume. **(A)** Correlation between the left frontalpole cortex and PSQI. **(B)** Correlation between the left frontalpole cortex and Sleep Quality. **(C)** Correlation between the left frontalpole cortex and Daytime Dysfunction. **(D)** Correlation between the left entorhinal cortex and Sleep Quality. **(E)** Correlation between the left entorhinal cortex and Sleep Duration. **(F)** Correlation between the right pericalcarine cortex and Sleep Latency. PSQI, Pittsburgh Sleep Quality Index.

A partial correlation analysis showed that the increase thickness of the left frontalpole cortex was positively correlated with the PSQI (***p*** = 0.038, **r**=0.27) and two sub-items (Sleep Quality, ***p*** = 0.04, **r**=0.27; Daytime Dysfunction, ***p*** = 0.037, **r**=0.28) in MMTS patients. Meanwhile, although the other two cortex were not significantly correlated with the PSQI, they were associated with several sub items respectively (left entorhinal cortex: Sleep Quality, ***p*** = 0.017, r=0.31; Sleep Duration, p =0.041, r=0.27; right pericalcarine cortex: Sleep Latency, p =0.036, r=0.27). Moreover, there were no correlations between the ISI scores and any increased cortex of the MMTS participants. These results were presented in **Figure 3**.

## 4 Discussion

Our study revealed significant increased cortical thickness in MMTS patients compared to MMT controls within the left entorhinal cortex, right pericalcarine cortex, and left frontalpole cortex, even after adjusting for key four covariates. Highlighted the potential neurobiological interactions between chronic methadone exposure, and sleep disruption.Notably, the left frontalpole cortex demonstrated a positive correlation with the PSQI and its subcomponents (Sleep Quality and Daytime Dysfunction). While the left entorhinal and right pericalcarine cortex alternations were not associated with the PSQI, they exhibited distinct correlations with specific PSQI sub-items (Sleep Quality/Duration and Sleep Latency, respectively), implying region-specific neurobiological contributions to different facets of sleep disruption. There were no correlations betwee all cortical alternations and the ISI. The absence of correlations with the ISI further highlighted the specificity of these structural changes to subjective sleep quality rather than insomnia severity. These findings aligned with emerging frameworks that positioned sleep as a regulator of neuroplasticity in addiction-related circuits (20, 21). Suggesting that sleep disturbances in the MMTS patients were associated with different cortical remodeling patterns, especially in areas crucial for addiction circuits and sleep regulation.

The cortical thickening observed in the left entorhinal cortex, right pericalcarine cortex, and left frontalpole cortex of MMTS patients contrasted with the neuroimaging patterns of the non-insomnia MMT group, suggesting that the pharmacological characteristics of methadone, combined with sleep disturbances, may disrupt sleep homeostasis, this disruption results in significant neural responses, consistent with previous studies (22). However, these results were contrasted with a study comparing MMT patients with opioid-naive individuals (23), our comparisons within MMTS emphasized specific regional hypertrophy. Unlike VBM studies that measure volume, our SBM approach specifically isolates cortical thickness changes, which may have reflected compensatory adaptation to prolonged μ-opioid receptor modulation under sleep disturbances. For example, methadone’s NMDA receptor antagonism might have reduced glutamate-mediated excitotoxicity, promoting synaptic preservation or remodeling in stress - susceptible areas (24, 25).

The findings on the alternations of the left entorhinal cortex in MMTS patients were consistent with a research of addiction memory but introudced dependent specificity of sleep disturbances. Similar results were seen in another study reporting the increased grey matter volume in entorhinal cortex of OUD patients after conventional treatment (non-methadone) compared to before treatment (26). Thus, it was worth noting that this structural plasticity (cortical thickness increase) might not only be a mere pathological compensation but also a potentially functional remodeling related to sleep disturbances. Additionally, our results of thicken entorhinal cortex corresponded to animal models, indicated that sleep disturbances enhanced synaptic plasticity in this circuit through BDNF-TrkB signaling (animal model sleep deprivation drove neural plasticity (27, 28). This model suggested a bidirectional mechanism between entorhinal cortex thickness and sleep disturbances: poor sleep might have exacerbated BDNF dysregulation in MMTS patients induced by methadone (29, 30), while increased cortex thickness might have disrupted sleep homeostasis by impairing hippocampal-entorhinal information transmission (31–33). Thus, it might form a vicious cycle, as increased thickness of entorhinal cortex might interfere sleep homeostasis, particularly slow-wave sleep (34, 35), a key medium for maintaining addiction memories (36), potentially increasing the risk of opioid relapse in MMTS patients.

The alternations of the right pericalcarine cortex introduced a new perspective to the pathophysiology of the MMTS. In previous studies on patients with insomnia alone or MMT patients, the pericalcarine cortex, a part of visual cortex, generally remained stable. However, a previous animal research found that opioids could affect the ability to synchronize circadian rhythms with the environmental light cycle in rats by acting on a specific subgroup of retinal cells: intrinsically photosensitive retinal ganglion cells (ipRGC), thus disrupting sleep/wake behavior (37). Therefore, we tried to apply this model to speculated that the increased thickness of the right pericalcarine cortex observed in MMTS patients might have been a result of methadone’s impact on ipRGCs and their related pathways, leading to sleep-wake homeostasis disruption in patients, manifested as prolonged sleep latency, which also explained the positive correlations between the increased thickness of the right pericalcarine cortex and sleep latency. Another possibility to explain the alternations in thickness of pericalcarine cortex was that the long half-life of methadone might disproportionately disrupt the melatonin secretion rhythm in MMTS patients, altering the light-carrying plasticity of the primary visual cortex. Evidence supported this speculation was that melatonin had played an important role in the circadian rhythm of pain, and μ-opioid receptor antagonists could disrupt the rhythm (38). Thus, methadone might have indirectly affected pain management and altered the melatonin secretion rhythm by acting as a μ-opioid receptor agonist. Moreover, sleep deprivation alone also interfered with melatonin secretion (39). Melatonin secretion disorders were related to cortical excitation-inhibition homeostasis, as confirmed in the practice of autism spectrum disorder (40) and schizophrenia (41). A previous double-blind, placebo-controlled and parallel-group study found that supplementing an appropriate amount of melatonin (10 milligrams per day before bedtime) during the MMT period in OUD patients could improve their subjective sleep quality, as well as depression and anxiety scores (42). Therefore, the current findings echoed some animal models, providing evidence of visual cortex abnormalities in OUD or MMT patients. These results might support future research to explore the role of the visual cortex in the treatment strategies for sleep disturbances in MMTS patients.

The increase thickness of left frontalpole cortex of MMTS patients in this study seemed to contradict the consistent conclusion of frontal lobe atrophy or even damage in OUD researches (43–45). However, it should be pointed out that these studies either used healthy controls or OUD pre-treatment as control groups, or did not consider the potential sleep disturbances as an independent role of in opioid guided neurometabolic models. After controlling for sleep factors, the current results suggested that sleep disturbances might play a key role in regulating the frontal lobe cortex neuroplasticity of the MMT. Consistent with this view, the frontal lobe was considered one of the key areas for regulating sleep, especially REM sleep (46). For healthy individuals without the MMT or USD, sleep deprivation usually led to negative impacts on cognition and decision-making, associated with frontal cortex damage (47). Despite the inconsistent findings reported in relevant studies, several lines of evidence supported the current observations. For example, increased excitability or volume of the frontal cortex due to sleep deprivation had been observed in animal models (48, 49), and human experiments had confirmed that sleep deprivation increased the intrinsic functional connectivity of the frontal cortex (50). Moreover, in the context of MMT, a reduction in a Fas-related protein induced apoptosis had been found to be associated with enhanced frontal cortex neuroplasticity (51). Although there was no direct evidence linking the Fas-related proteins to sleep disturbances, a study on the hypnotic mechanism of ketamine (an antidepressant and sedative) in mice found that ketamine might reduce the relative number of Fas-related proteins, thereby abnormally increasing neuroplasticity (52). Considering that this study strictly controlled for sleep disturbances or depression related medicine in all MMT and MMTS patients in the past two years, but did not recognize the medicine history before two years by case tracking, the current findings might be also related to the long-term efficacy of ketamine or other medicine that acted on Fas-related proteins than methadone. The correlations between the increased thickness of the left frontalpole cortex and PSQI and its sub-items especially the Daytime Dysfunction indicated that this “overgrowth” reflected inefficient neural resource allocation in the MMTS patients. Compensatory synaptic proliferation in the frontal cortex might initially enhance cognitive control in cravings and be detrimental to impulse inhibition (53), thus eventually it could overwhelm metabolic capacity and increase the risk of treatment interruption and relapse in MMTS patients.

Building on these region-specific structural adaptations linked to synaptic plasticity, we would further explore how sleep disturbances might drive cortical remodeling through two main pathways in MMTS patients. On one hand, as the above mentioned, considering that methadone acted as both a μ-opioid receptor agonist and an NMDA receptor antagonist (3), the presence of sleep disturbances might synergistically alter the cortical homeostasis and enhance neuroplasticity of MMTS patients in the context of MMT (54). On the other hand, sleep disturbances could alter cortical homeostasis through neuroinflammation (55). It was worth noting that sleep disturbances or sleep deprivation have been shown to be associated with systemic inflammatory markers, such as CRP and IL-6 (56). And further research suggested that sleep deprivation could activate inflammatory pathways, leading to glial proliferation and morphological changes in microglia and astrocytes (57). The dynamic changes in glial cells could potentially account for the observed cortical thickening in the study, contrary to the anticipated thinning. Moreover, another study found that sleep deprivation might reverse neuroplasticity by inhibiting immune and inflammatory pathways, thereby alleviating depressive-like behaviors in mice (58). However, the specific mechanisms linking sleep disorders and inflammation in MMT patients remained unclear at present, and whether inflammation-related signaling pathways were fully/partially activated or inhibited, or whether there was synergy/antagonism between pathways, required further research in the future.

There were several limitations in this study. Firstly, although the sample size met the requirements of *post-hoc* power analysis, a relatively small size might limit the generalizability of the results. Larger cohorts were needed to verify the observed cortical thickness differences and explain the potential heterogeneity of individual responses to methadone and sleep disturbances. Secondly, the cross-sectional design precluded causal inferences about whether sleep disturbances directly drove cortical remodeling or vice versa, or whether both them were affected by unmeasured confounding factors. Longitudinal studies of tracking cortical changes as well as sleep quality and treatment records were crucial for clarifying the temporal relationships. Next, reliance on self-reported sleep quality assessments (PSQI and ISI) might introduce potential subjective bias, objective measurements such as PSG records could strengthen the future research. Fourth, given the exploratory nature of this study, strict multiple comparison corrections (e.g., Bonferroni) were not applied to avoid Type II errors. While this strategy aids in identifying potential biomarkers, it implies a risk of false positives; thus, these findings should be considered preliminary and require validation in independent cohorts. Finally, although major comorbidities of mental illness were excluded, there was no strict control over a medication history of more than two years which may be related to the sleep disturbances or of the MMTS population, and may confuse the observed cortical alternations and associations.

## 5 Conclusions

In conclusion, this study provided neuroimaging evidence linking sleep disturbances to specific cortical thickening in the left entorhinal, right pericalcarine, and left frontalpole cortices in MMT patients. Sleep disturbances might change the neuroanatomical homeostasis in MMT patients and could potentially serve as a significant feature for identifying high-risk groups. Therefore, this work emphasized the necessity of incorporating sleep-targeted interventions into MMT protocols. Further longitudinal studies with multimodal imaging, objective sleep records and clinical data were crucial for confirming the prognostic value of these cortical changes and optimizing personalized treatment strategies for MMTS patients.

## Data Availability

The raw data supporting the conclusions of this article will be made available by the authors, without undue reservation.
